# Chikungunya Virus Exploits miR-146a to Regulate NF-κB Pathway in Human Synovial Fibroblasts

**DOI:** 10.1371/journal.pone.0103624

**Published:** 2014-08-01

**Authors:** Sakthi Priya Selvamani, Ritu Mishra, Sunit K. Singh

**Affiliations:** Laboratory of Neurovirology and Inflammation Biology, CSIR-Centre for Cellular and Molecular Biology (CCMB), Hyderabad, India; Singapore Immunology Network, Agency for Science, Technology and Research (A*STAR), Singapore

## Abstract

**Objectives:**

Chikungunya virus causes chronic infection with manifestations of joint pain. Human synovial fibroblasts get infected with CHIKV and could lead to pro-inflammatory responses. MicroRNAs have potentials to regulate the gene expression of various anti-viral and pro-inflammatory genes. The study aims to investigate the role of miR-146a in modulation of inflammatory responses of human synovial fibroblasts by Chikungunya virus.

**Methods:**

To study the role of miR-146a in CHIKV pathogenesis in human synovial cells and underlying inflammatory manifestations, we performed CHIKV infection in primary human synovial fibroblasts. Western blotting, real-time PCR, luciferase reporter assay, overexpression and knockdown of cellular miR-146a strategies have been employed to validate the role of miR-146a in regulation of pro-inflammatory NF-κB pathway.

**Results:**

CHIKV infection induced the expression of cellular miR-146a, which resulted into down-regulation of TRAF6, IRAK1, IRAK2 and increased replication of CHIKV in human synovial fibroblasts. Exogenous expression of miR-146a in human synovial fibroblasts led to decreased expression of TRAF6, IRAK1, IRAK2 and decreased replication of CHIKV. Inhibition of cellular miR-146a by anti-miR-146a restored the expression levels of TRAF6, IRAK1 and IRAK2. Downregulation of TRAF6, IRAK1 and IRAK2 led to downstream decreased NF-κB activation through negative feedback loop.

**Conclusion:**

This study demonstrated the mechanism of exploitation of cellular miR-146a by CHIKV in modulating the host antiviral immune response in primary human synovial fibroblasts.

## Introduction

Chikungunya disease is an arboviral disease caused by CHIKV. CHIKV is transmitted by *Aedes* species of mosquitoes, and it has emerged as a major public health problem in many parts of the world [1], [2]. CHIKV is known as an arthritogenic virus belonging to *Togaviridae* family of genus *Alphavirus* [3], [4]. CHIKV has a single stranded RNA genome of positive polarity and of approximately 12 kb in size.

CHIKV was first reported in East Africa in 1952 [5], [6]. During acute infection, CHIKV is known to infect the fibroblast cells of muscle, joint synovium and skin [7], [8], affecting wrists, fingers, elbows, toes, ankles and knees causing severe pain collectively known as polyarthralgia and polyarthritis [2], [9]. Biopsy from CHIKV infected patients showed high viremia in isolated fibroblasts [10]. Persistence of CHIKV in tissues and organs has been reported in various animal models [8], [11], [12]. Chikungunya fever is mostly characterised by headache, nausea, polyarthralgia, fever, myalgia and rashes [13], [14]. CHIKV has been reported to trigger apoptosis through intrinsic and extrinsic pathways in primary human synovial fibroblasts [15]. CHIKV infected fibroblasts exhibit perturbation in type I interferons production *in vitro* and *in vivo* studies [16]. The expression of type I interferons as well as other pro-inflammatory cytokines in CHIKV infected fibroblasts have been demonstrated through MDA5 and RIG-I pathway [7]. In previous studies, CHIKV has been reported to modulate the interferon response in fibroblast cell lines by inhibiting the nuclear translocation of phospho STAT1 [17].

However; microRNA (miRNA/miR) mediated regulation of antiviral response in primary human synovial fibroblasts upon CHIKV infection has not been investigated so far. miRNAs are small non-coding RNAs, 19–22 nucleotides in length, leads to post transcriptional gene regulation by binding to complementary sites in 3′UTR of target gene via their seed region [18], [19]. MicroRNAs play important roles in regulation of various biological processes such as inflammation, infection, immune response and tumorigenesis etc [20], [21]. Viruses are known to modulate the expression pattern of cellular miRNAs in host cells [22]. The involvement of miR-146 has been reported in cellular host immune responses during microbial infections [23], [24], [25]. The induction of miR-146a is NF-κB dependent and directly down regulates the signal transducers like TNF receptor-associated factor 6 (TRAF6) and IL-1 receptor associated kinase 1 (IRAK1). Therefore miR-146a suppresses the NF-κB signalling and suppresses the inflammatory response via negative feedback loop [25],[26]. The alterations in the expression levels of miR-146a have been reported in various inflammatory conditions [27], [28], [29]. Taganov *et al.*, 2006; reported elevated levels of miR-146a expression in THP-1 cells by LPS, IL-1β and TNFα stimulation [25]. Promoter region of miR-146a has several NF-κB binding sites, which suggests the NF-κB dependent induction of miR-146a expression [25], [30]. TRAF6 and IRAK1 are key adaptor molecules in TIR signalling pathway, which have been shown to be suppressed during induction of miR-146a leading to the suppression of IL-6, IL-8, IL-1β and TNFα due to impaired NF-κB activity [31], [32], [33], [34].

Increased expression levels of miR-146a have been previously reported in synovial tissues of Rheumatoid Arthritis (RA) patients [35], [36], [37]. CHIKV is known to be arthritogenic and their pathological manifestations have been intricately related to overactivation of host inflammatory responses. Therefore, we further investigated the role of miR-146a in CHIKV infected human synovial fibroblasts. miR-146a is known to play important role during inflammatory responses. The gene expression studies in RA and CHIKV arthritis patients show a significant overlap in the expression profiling of inflammatory genes [38].

The role of miRNA in Alphavirus infection has not been understood so far. This study is focused to understand the changes in the expression levels of miR-146a, mechanism of regulation of the NF-κB pathway and pro-inflammatory responses in primary human synovial fibroblasts upon CHIKV infection.

## Materials and Methods

### Cell culture

Primary human synovial fibroblasts isolated from normal knee synovium of 32 years old Caucasian male were purchased from Asterand (Asterand, Michigan, USA). Synovial fibroblasts were cultured in Dulbecco’s Modified Eagle’s Medium (DMEM) (Invitrogen) supplemented with 20% fetal bovine serum, 100 U/ml of penicillin and 100 µg/ml streptomycin (Invitrogen). For reporter assay HEK 293T cells were grown in DMEM (Invitrogen) supplemented with 10% fetal bovine serum, 100 U/ml of penicillin and 100 µg/ml streptomycin (Invitrogen). Vero cells were cultured in DMEM media supplemented with 10% fetal bovine serum, 100 U/ml of penicillin and 100 µg/ml streptomycin. All the cultures were maintained at 37°C incubator with a constant supply of 5% CO_2_.

### Virus propagation and Infection

CHIKV (Ross strain, E1:A226) was obtained from Prof. Duane Gubler (Emerging Infectious Disease Program, Duke-NUS Medical School, Singapore) as a kind gift. The virus was propagated by a single passage on Vero cells. After 72 hours of inoculation, culture supernatants were collected and centrifuged at 3500×g for 30 min to remove cell debris and stored at −80°C.

Human synovial fibroblasts were infected at a Multiplicity of Infection (MOI) 2 in serum free DMEM medium for 3 hour at 37°C CO_2_ incubator. After 3 hours, cells were washed with 1X PBS and supplemented with complete media containing 20% serum and harvested at 32 hours post infection for RNA isolation and protein analysis.

### Viral plaque assay

To determine viral titer, viral plaque assay was performed. Vero cells were grown to 90% confluency in 6-well culture plates and serially diluted virus (10^−3^ to 10^−10^) in serum free DMEM were added to the cells. Followed by 1–2 hour of virus absorption at 37°C, the media was removed, washed with 1X PBS (Phosphate Buffered Saline). Cells were then covered by an overlay of low melting agarose (2%) (Invitrogen) and incubated at 37°C humidified incubator for 3 days. The cells were fixed by addition of 10% formaldehyde at room temperature for 4 hours followed by removal of agarose overlay and staining of plaques by 0.1% crystal violet stain. The virus titre was determined as plaque forming units (PFU) per millilitre of the supernatant.

### RNA isolation and miRNA assay

Total RNA including the microRNA fractions was isolated by using miRNeasy kit (Qiagen; Germany). cDNA synthesis of miRNA has been performed by TaqMan reverse transcription kit (Applied Biosystems) using primers specific to miRNA as per manufacture’s instruction. Briefly, thermal incubation for cDNA synthesis were as follows: 16°C for 30 min, 42°C for 30 min, and 85°C for 5 min. Quantitative Real Time PCR (qRT-PCR) for miRNA assay was performed using miRNA-specific TaqMan probes and universal PCR master mix (Applied Biosystems). Thermal incubation for real time PCR was as follows: 95°C for 10 min, followed by 40 cycles of 95°C for 15 seconds and 60°C for 60 seconds. All the quantitative real time PCR experiments have been run on thermal cycler ABI 7900 and Roche Light Cycler 480. To normalize miR-146 expression, RNU24 expression levels were checked in all the experiments and fold changes in miR-146 were calculated by using standard ΔΔ ct method.

### Reverse transcription and PCR

cDNA synthesis of isolated RNA has been performed by reverse transcription kit, Superscript II (Invitrogen) as per the manufacturer’s protocol. Thermal incubations for cDNA synthesis were as follows: 65°C for 5 min, 25°C for 10 min, 42°C for 50 min, 70°C for 10 min. Finally the products were treated with RNase H for 20 min at 37°C for removal of residual RNA. CHIKV infection in synovial fibroblasts was confirmed by PCR with primers specific for viral 3′UTR region in infected samples. Primers used were as follows: β-actin Forward 5′TCATGAAGTGTGACGTGGAC3’, Reverse 5′CAGGAGGAGCAATGATCTTGAT3’, CHIKV Forward 5′GGAAGCTGAGATAGAAGTTGAAGG3’ and CHIKV Reverse 5′CATCTCCTACGTCCCTGTGGGTT3’). Thermal cycles were as follows: 98°C for 30 s, followed by 40 cycles of 98°C for 10 s, 55°C for 30 s, 72°C for 50 s and a final extension of 72°C for 10 min.

### Cell lysate and Western Blotting

Cell lysates were prepared with RIPA buffer (150 mM NaCl, 1% NP-40, 50 mM Tris-HCl, pH 7.5, 0.1% SDS and 0.5% sodium deoxycholate supplemented with 1X protease inhibitor cocktail) for Western blotting. Protein concentrations were determined by BCA protein assay kit (Novagen). Protein samples were run on 12% SDS gel and transfer was performed at 100 V for 2 hour onto PVDF membrane (Millipore). Membranes were blocked in 5% skimmed milk. Membranes were incubated with primary antibody at 4°C for overnight. Membranes were washed with three washes of 15 min each with TBST buffer. HRP conjugated secondary antibody was incubated onto membrane for 1 hour and followed by three washes for 15 min each with TBST buffer. The membranes were developed by using Super-Signal developing reagent (Pierce). Antibodies, anti-TRAF6, anti-IRAK1, anti-IRAK2, anti-phospho NF-κB p65, anti-NF-κB p65 (Cell Signaling Technology) and anti-β-tubulin (Abcam) have been used in the study. Western blots band intensities were quantified by using ImageJ software and normalized by β-tubulin image density.

### MicroRNA overexpression

Synovial fibroblasts were seeded in T-25 flasks and over expression of miRNA-146a mimics were performed at 70% confluent monolayer. For over expression of miR-146a, RNA oligos were used in the study (miR-146a, Table-1). As a negative control, scrambled oligo sequence of miR-146a was used (scrambled miR-146a, Table-1). The sequence of mature miR-146a was retrieved from miRBase and commercially synthesized by local supplier (Bioserve Ltd, Hyderabad, India). Transfection mix was prepared in commercial low serum media Opti-MEM (Invitrogen) and transfection was done in antibiotic-free media with 100 picomoles of miR-146a mimic with Lipofectamine 2000 (Invitrogen) according to the manufacturer’s protocol. 100 picomoles of scrambled miR-146a mimic were transfected into synovial fibroblasts as negative control. Transfection efficiency was monitored by visualization of Green Fluorescent Reporter (GFP) which was used as positive control for transfection procedures. Cells were harvested for RNA isolation and protein lysate preparation 48 hours post transfection. miR-146a over expression was confirmed by qPCR using TaqMan assay specific to miR-146a. Expression levels of the target proteins in transfected cells were analyzed by Western blotting by using the corresponding antibodies.

**Table 1:** MicroRNA sequences.

Name of RNA Oligos	Sequence
miR-146a	UGAGAACUGAAUUCCAUGGGUU
Scramble miR-146a	GGAUGUAUGCUGCUGCUAAUAA

### Anti-miR (miRNA inhibitor) transfection

Synovial fibroblasts were transfected with 100 picomoles of anti-miR-146a (Ambion) and Cy3-labeled control anti-miR (Ambion) with Lipofectamine 2000 as per the manufacturer’s protocols. Efficiency of transfection was monitored by visualizing the fluorescence of Cy3-labeled control anti-miR. Cells were harvested for RNA isolation and protein lysate preparation 48 hours post transfection. Knockdown of miR-146a in anti-miR transfected cells were confirmed by quantitative real time PCR using TaqMan assay specific to miR-146a. Protein expression levels of miR-146a targets were analysed in the transfected cells by Western blotting with the corresponding antibodies.

### NF-κB Luciferase reporter assay

For reporter assay, HEK 293T cells were seeded in 6-well culture dishes until ∼70% confluency and transfected with 2 µg of NF-κB -FLuc plasmid (a kind gift from Dr. Adolfo Garcia Sastre, Mount Sinai School of Medicine, New York, USA) using Lipofectamine 2000 as per the manufacturer’s protocol. For infection experiments, 6–8 hours post transfection, cells were infected with CHIKV at MOI 2 and harvested 32 hours post infection to measure luciferase activity. For microRNA over expression experiments, cells were co transfected with reporter clone of NF-κB and miR-146a/scramble miR-146a mimic and harvested 48 hours post transfection to measure the luciferase activity. In anti-miR experiment, 24 hour post transfection of reporter plasmids along with anti-miR-146a, cells were infected with CHIKV and lysates were prepared for luciferase activity. Luciferase activity was determined as relative light unit by using luciferase assay kit (Promega) according to the manufacturer’s protocol and normalized with β-galactosidase expression by β-galactosidase assay kit (Promega).

### Statistical analysis

Results are expressed as mean ± SEM from three independent biologically repeated experiments. Level of significance (p values) was determined by Student’s *t* test between treated group versus untreated (control) group; considering *p*≤0.05 as significant in two-tailed student’s t-test by applying paired/equal/unequal variance. The fold changes of miR-146a in treated groups have been compared with untreated controls by using ΔΔ ct methods. All the comparisons and statistical analysis have been done in Microsoft excel.

## Results

### CHIKV infection increases the expression levels of miR-146a in primary human synovial fibroblasts

**Figure 1:**
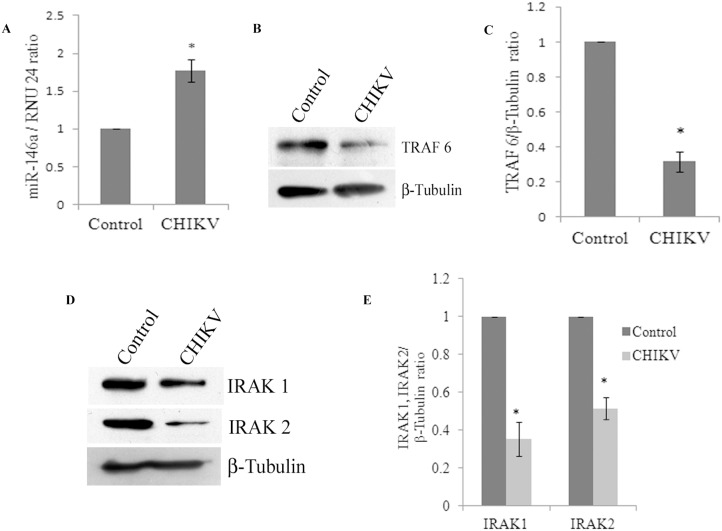
CHIKV infection increases miR-146a expression and decreases TRAF6, IRAK1, and IRAK2 in primary human synovial fibroblasts. Expression of miR-146a was increased upon CHIKV infection. (A). Human primary synovial fibroblasts were infected with CHIKV at MOI of 2 and cells were harvested 32 hours post infection for RNA isolation and protein lysate preparation. Expression levels of miR-146a were determined by qRT-PCR with TaqMan primers and probes specific for miR-146a. Expression level of RNU 24, an endogenous control, has been used as normalizer and the results are shown as fold changes compared to controls. (B) Western blot analysis showing decrease in the protein expression levels of TRAF6 in CHIKV infected primary human synovial fibroblasts. (C) Graph bars are showing densitometry analysis of TRAF6 normalized with housekeeping gene β-tubulin by ImageJ software. (D). Western blot analysis showing decrease in protein expression levels of IRAK 1 and IRAK 2 in CHIKV infected primary human synovial fibroblasts compared to uninfected controls. (E) Graph bars representing densitometry analysis for expression levels of IRAK1 and IRAK2 normalized with β-tubulin. All the experiments were independently repeated three times and shown as mean ± SEM. * above bars are representing the p value≤0.05 as level of significance, n = 3 (*for p value≤0.05).

The roles of microRNAs in the modulation of immune responses have been reported in various viral infections [23], [39], [40]. miR-146a have been reported to play important roles in the regulation of pro-inflammatory responses [36]. We investigated the potential perturbation of miR-146a in primary human synovial fibroblasts after CHIKV infection. Synovial fibroblasts were infected with CHIKV at MOI of 2 for 32 hours. Changes in target protein expression levels were initially measured after 6, 12, 24 and 32 hours of CHIKV infection. However, significant alterations in the expression pattern of specific target genes (TRAF6, IRAK1 and IRAK2) were observed only after 32 hours of infection. Therefore, further studies were carried out at 32 hours post-infection. The cellular expression levels of miR-146a were determined by real time PCR using TaqMan primers and probes specific for miR-146a. The expression levels of miR-146a in CHIKV infected primary human synovial fibroblasts were 1.77 fold higher, compared to uninfected controls (Figure-1A).

### CHIKV infection down regulates TRAF6, IRAK1 and IRAK2 expression level in primary human synovial fibroblasts

TRAF6, IRAK1 and IRAK2 play major roles in regulation of immune responses during viral infections. In CHIKV infected primary human synovial fibroblasts, the expression levels of target genes of miR-146a such as TRAF6, IRAK1 and IRAK2 were analyzed after 32 hours of CHIKV infection. Significant downregulation, (69% decrease in TRAF6, 65% decrease in IRAK1 and 49% decrease in IRAK2) (p≤0.05) at the protein expression levels of TRAF6, IRAK1 and IRAK2 was observed in CHIKV infected human synovial fibroblasts (Figure-1B, C, D, E).

### miR-146a overexpression decreases TRAF6, IRAK1 and IRAK2 protein expression levels

**Figure 2:**
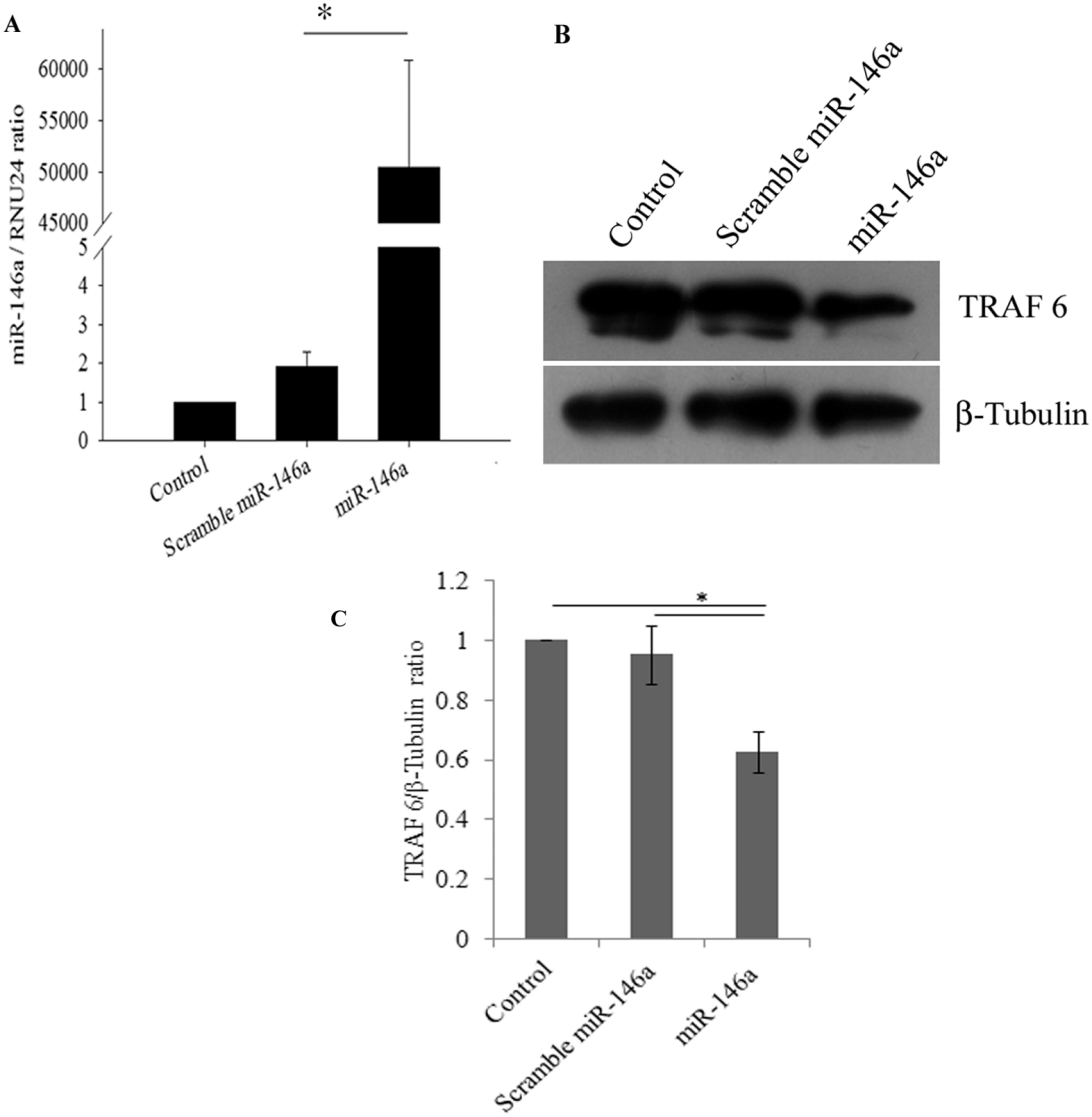
miR-146a overexpression decreases TRAF6 protein expression levels in primary human synovial fibroblasts. Overexpression of miR-146a suppresses protein expression levels of TRAF6. (**A**) miR-146a expression levels were determined by qRT-PCR with TaqMan primers and probes specific to miR-146a. Scramble and miR-146a transfected primary human synovial fibroblasts were harvested 48 hours post transfection and fold change of miR-146a in overexpressed cells were significantly higher with respect to scrambled miR-146a (*p<0.05). (**B**) Western blot analysis showing significant decrease in the protein expression levels of TRAF6 in miR-146a overexpressed primary human synovial fibroblasts compared to controls and scrambled miR-146a. (**C**) The graph bars are representing densitometry analysis of TRAF6 expression levels normalized with β-tubulin. All the experiments have been done three times and results are shown as mean ± SEM. (* for p value≤0.05).

**Figure 3:**
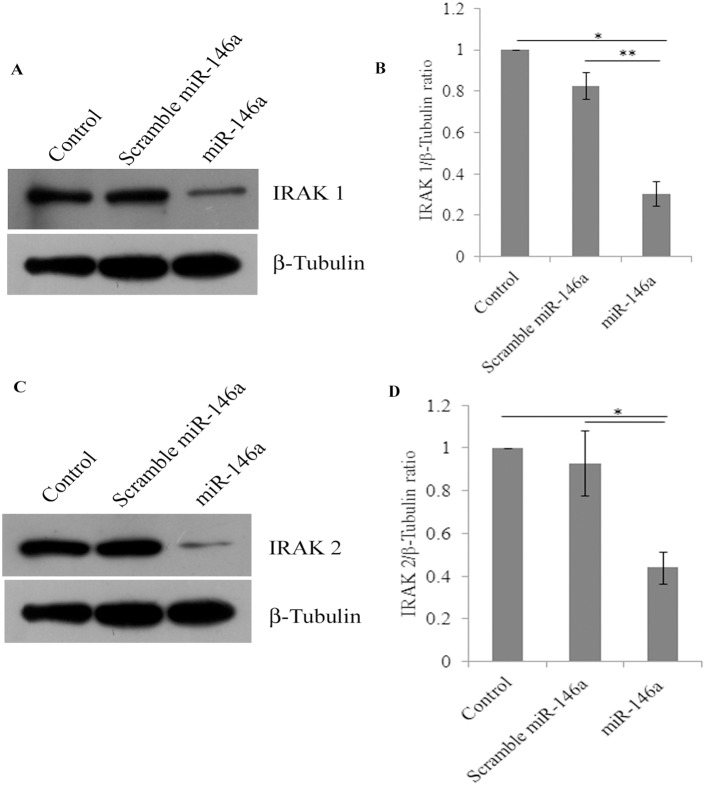
miR-146a overexpression decreases IRAK1 and IRAK2 protein expression levels in primary human synovial fibroblasts. (A) Western blot analysis showing down regulation of IRAK1 at the protein expression level in miR-146a over expressed primary human synovial fibroblasts. (B) Densitometry analysis of IRAK1 expression levels normalized with β-tubulin. (C). Western blot analysis showing decreased expression of IRAK2 at the protein levels in miR-146a overexpressed primary human synovial fibroblasts. (D) Densitometry analysis of IRAK2 protein expression levels upon normalization with β-tubulin. Experiments were repeated three times and results shown as mean ± SEM. (* for p value≤0.05, ** for p value≤0.005).

In primary human synovial fibroblasts, the mimics of miR-146a were transfected exogenously to analyse the effect on the protein expression levels of target genes. miR-146a mimics were transfected at the concentration of 100 picomoles and scrambled miR-146a were transfected as a negative control with same concentration of 100 picomoles. Scrambled miR-146a were transfected in primary human synovial fibroblasts to demonstrate the specificity of miR-146a in regulating the expression levels of target genes through the binding of the complementary sequences of the seed region of miRNA with the 3′UTR of target mRNA. The expression levels of miR-146a were significantly higher upto 50,000 fold in overexpressed cells (Figure-2A) (p≤0.05); compared to cells transfected with scrambled miR-146a (p≤0.05). Significant down regulation (38% decrease in TRAF6, 70% decrease in IRAK1 and 56% decrease in IRAK2) was observed in protein expression levels of TRAF6, IRAK1, and IRAK2 (Figure-2B, Figure-3) in miR-146a overexpressed synovial fibroblast cells. Transfection with scramble miR-146a did not show any significant change in the protein expression levels of TRAF6, IRAK1, and IRAK2.

### Anti-miR-146a transfection rescues the expression levels of TRAF6, IRAK1, and IRAK2 in CHIKV infected human synovial fibroblasts

**Figure 4:**
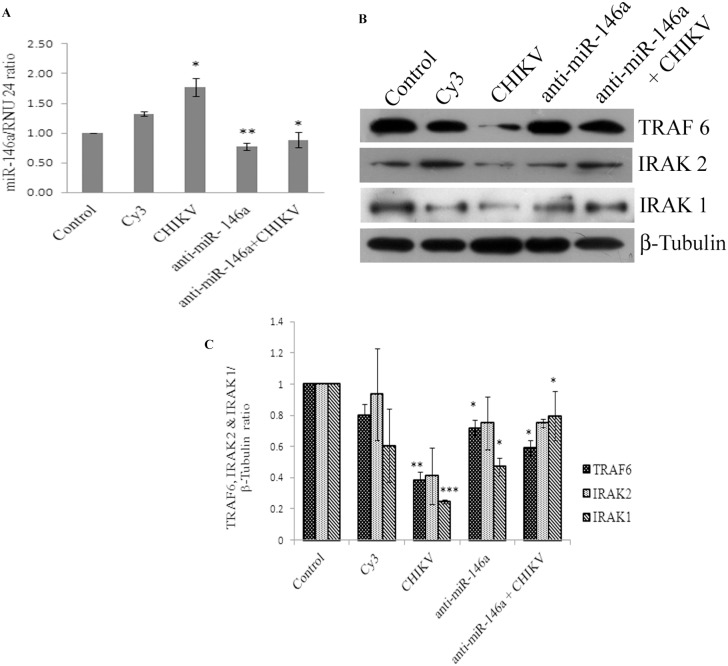
Anti-miR-146a rescues the target gene expression levels. Anti-miR-146a suppresses the miR-146a levels and rescues target gene expression levels. (**A**) The graph bars are showing a significant decrease in the expression of cellular miR-146a after transfection with anti-miR-146a (miR-146a inhibitors) compared to CHIKV infection (*≤0.05). Cy3 labelled controls anti-miR was used to check the transfection efficiency. RNU24 expression levels have been used as normalizer and fold change in miR-146a has been calculated by ΔΔct methods. (**B**) Western blot images showing the rescued expression of TRAF6, IRAK1 and IRAK2 due to knockdown of cellular miR-146a, 48 hours post anti-miR-146a transfection. (**C**) The graph bars representing densitometry analysis of western blot images for TRAF6, IRAK1 and IRAK2 normalized with β-tubulin. All the experiments were repeated in three biological replicates and represented as mean ± SEM. * above bars are representing the p value≤0.05 as level of significance, n = 3. (* for p value≤0.05, ** for p value≤0.005, *** for p value≤0.001).

The knockdown studies of miR-146a were performed to determine the direct regulatory role of miR-146a on the expression levels of TRAF6, IRAK1 and IRAK2 and further to demonstrate the potential of anti-miR-146a in rescuing the expression levels of the target genes. Cy3-labelled control anti-miR was used as a negative control as well as to visualize the transfection efficiency. These are scrambled RNA oligo sequences which are not possessing any complementary binding region towards any of hosts mRNA unlike miRNA-146a inhibitor which has a potential complementary region for seed sequences of cellular miR-146a. Synovial fibroblasts transfected with anti-miR-146a showed a significant suppression of cellular miR-146a (Figure-4A). Primary human synovial fibroblasts having reduced levels of miR-146a by anti-miR application were infected with CHIKV and expression levels of TRAF6, IRAK1 and IRAK2 were analysed. Expression levels of TRAF6, IRAK1 and IRAK2 (Figure-4B, C) were rescued significantly in human synovial fibroblasts transfected with anti-miR-146a. Synovial fibroblasts transfected with anti-miR-146a followed by CHIKV infection showed the expression patterns of miR-146a close to controls (Figure-4A). The expression levels of TRAF6 and IRAK1 proteins were rescued significantly and their sustained expression levels (p≤0.05) were observed even after CHIKV infection in primary human synovial fibroblasts (Figure-4B, C). As compared to CHIKV mediated downregulation of TRAF6 and IRAK1, anti-miR-146a transfected cells show significant rescue (p≤0.05) in their expression level. This observation demonstrated that CHIKV modulates the expression levels of miR-146a, which ultimately suppresses the expression of target genes in primary human synovial fibroblasts and such effect can be effectively counter-balanced by suppressing the cellular levels of miR-146a through microRNA inhibitors.

### CHIKV suppresses NF-κB activation in primary human synovial fibroblasts

**Figure 5:**
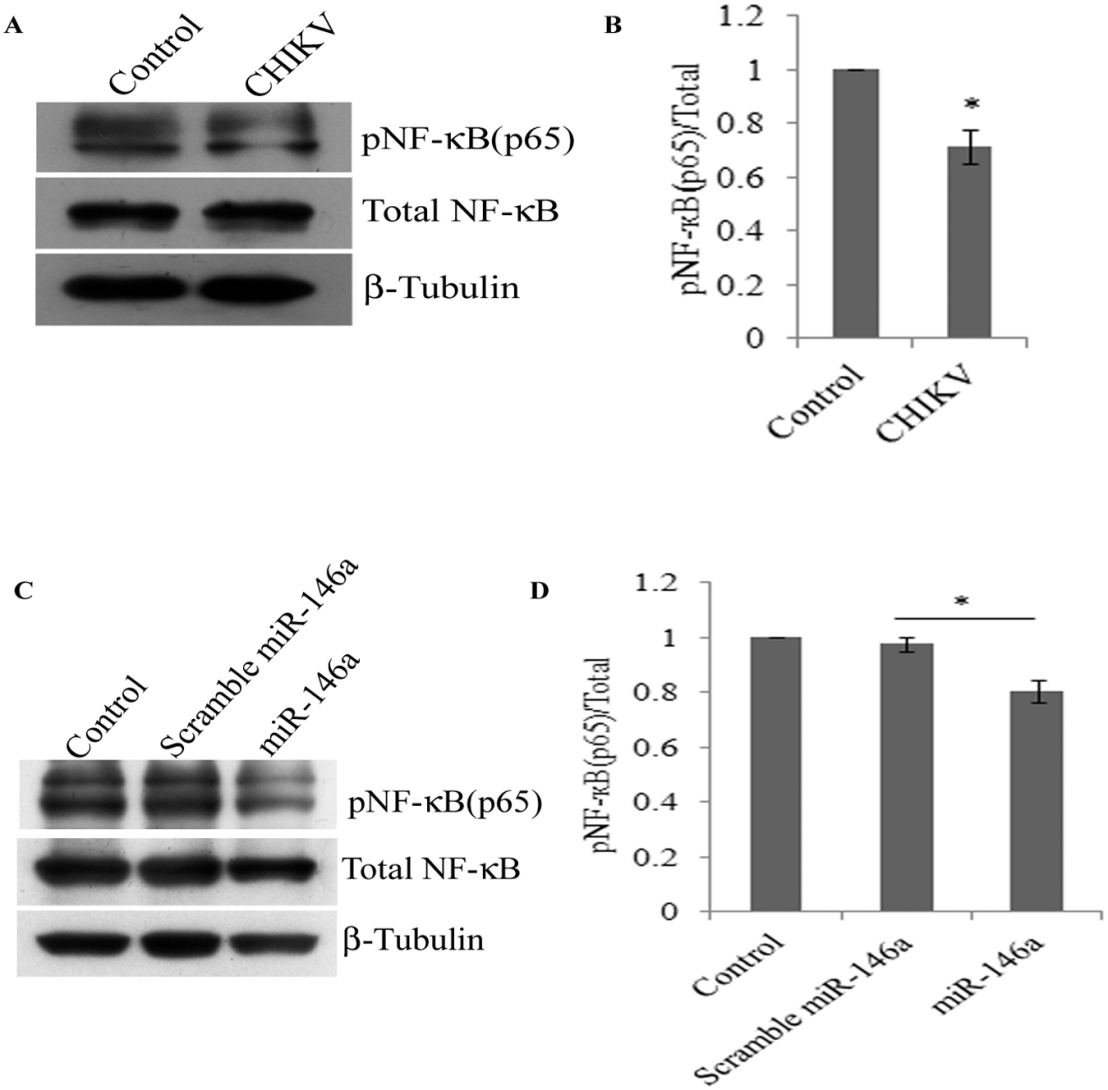
Induced expression of miR-146a decreases NF-κB activation. CHIKV infection decreases activation of NF-κB in negative feedback loop. (**A**). Western blot image showing decreased phosphorylation of NF-κB (p65), phospho NF-κB (p65) and total NF-κB (p65) after CHIKV infection in primary human synovial fibroblasts. (**B**) Densitometry analysis shown as graph bars for protein expression levels of p- NF-κB (p65) and total NF-κB (p65) normalized with β-tubulin. (**C**) Western blot analysis of p- NF-κB (p65) and total NF-κB (p65) protein expression levels in primary human synovial fibroblasts transfected with 100 pm scrambled and 100 pm miR-146a mimics. Increase in miR-146a significantly decreases p- NF-κB (p65) levels compared to controls and scramble miR-146a. (**D**) The graph bars representing densitometry of protein expression levels of p- NF-κB (p65) and total NF-κB (p65) after normalization with β-tubulin. Three independent experiments were performed and data is presented as mean ± SEM. *p<0.05.

Regulation of miR-146a is NF-κB dependent and induced by NF-κB activation; which can further down regulate TRAF6, IRAK1 and IRAK2. In this negative feed-back loop, NF-κB is a downstream nuclear executor; which regulates the transcription of various pro-inflammatory cytokines. We checked whether the elevated levels of cellular miR-146a upon CHIKV infection can suppress the expression and phosphorylation of NF-κB protein subunits (p65) in primary human synovial fibroblasts. 30% decrease in phosphorylation of NF-κB (p65) was observed (p≤0.05) in CHIKV infected synovial fibroblasts; compared to controls (Figure-5A).

### miR-146a overexpression decreases NF-κB activation

Perturbation in TRAF6, IRAK1 and IRAK2 expression levels and their downstream effects on NF-κB activation was investigated in primary human synovial fibroblasts through overexpression of miR-146a exogenously. The primary human synovial fibroblasts were transfected with scrambled and miR-146a mimics. Phosphorylation of NF-κB (p65) was significantly decreased after miR-146a overexpression as compared to the cells transfected with scramble miR-146a (Figure-5C).

**Figure 6:**
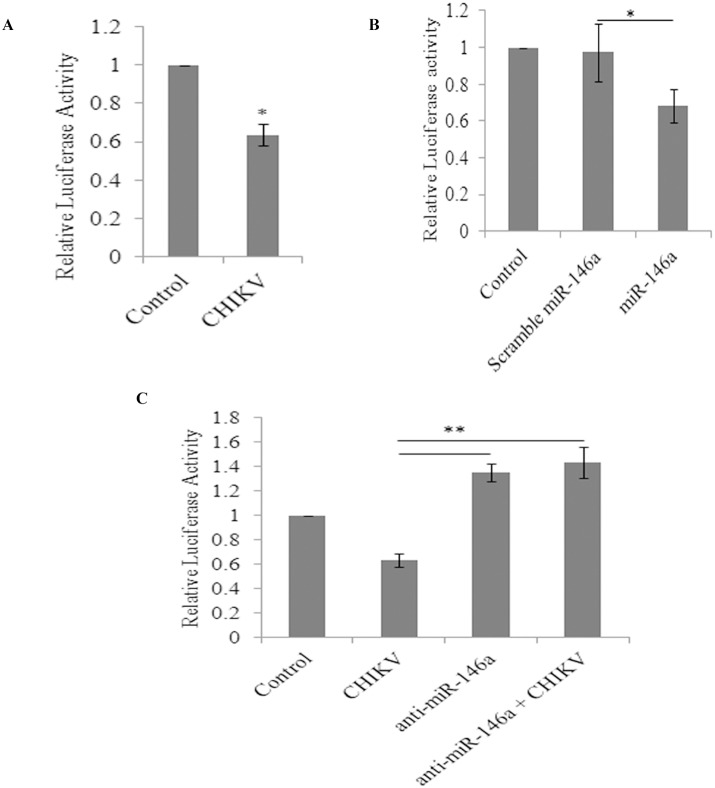
CHIKV suppresses **NF-κB** activity through miR-146a. Increase in miR-146a expression levels suppresses the activation of NF-κB. 2 µg of NF-κB -FLuc plasmid was co-transfected with 700 ng of pCMV-β-gal plasmid in HEK 293 T cells for reporter assay. 6–8 hours post transfection, cells were infected with CHIKV (MOI 2) and harvested 32 hours post infection for Luciferase assay. (**A**) The graph bars are representing relative luciferase activity of NF-κB in control and CHIKV infected primary human synovial fibroblasts after normalization with β-galactosidase expression units showing decreased NF-κB activity. (**B**) The graph bars representing relative luciferase units in miR-146a overexpression. Transfection of miR-146a decreases NF-κB activity compared to controls and scramble miR-146a. (**C**) HEK cells were infected with CHIKV after the 24 hours of anti-miR-146a transfection. Anti-miR-146a transfection rescues the activity of NF-κB even in cells infected with CHIKV. The graph bars are showing elevated luciferase activity in anti-miR-146a and anti-miR-146a+CHIKV infection. All experiments were performed in biological triplicates and data represented as mean ± SEM. (*for p value≤0.05, **for p value≤0.005).

The effect of CHIKV infection as well as miR-146a overexpression on NF-κB promoter activity was also validated by luciferase reporter assay in HEK-293 T cells. NF-κB promoter activity was decreased by 38% (p≤0.05) upon CHIKV infection as shown by decrease in luciferase activity (Figure-6A). Similarly, over expression of miR-146a also showed 30% decrease in NF-κB promoter activation; compared to scramble miR-146a transfected cells; which indicated the regulatory role of miR-146a in CHIKV mediated suppression of NF-κB promoter activation (Figure-6B).

NF-κB promoter activation was also analysed in anti-miR-146a transfected cells to study the rescued effect effects of TRAF6, IRAK1 and IRAK2 expression levels on NF-κB activation. NF-κB promoter activity was checked in another experimental set up of anti-miR-146a transfection followed by CHIKV infection in primary human synovial fibroblasts. Luciferase activity revealed significantly sustained activity of NF-κB (71% increase in anti-miR-146a transfected cells and 79% increase in anti-miR-146a+CHIKV transfected cells) (p≤0.005), 24 hours post transfection of anti-miR-146a followed by CHIKV infection (Figure-6C). These observations supported the negative regulatory role of miR-146a upon NF-κB activity.

### Overexpression and knockdown of miR-146a influences CHIKV replication

**Figure 7:**
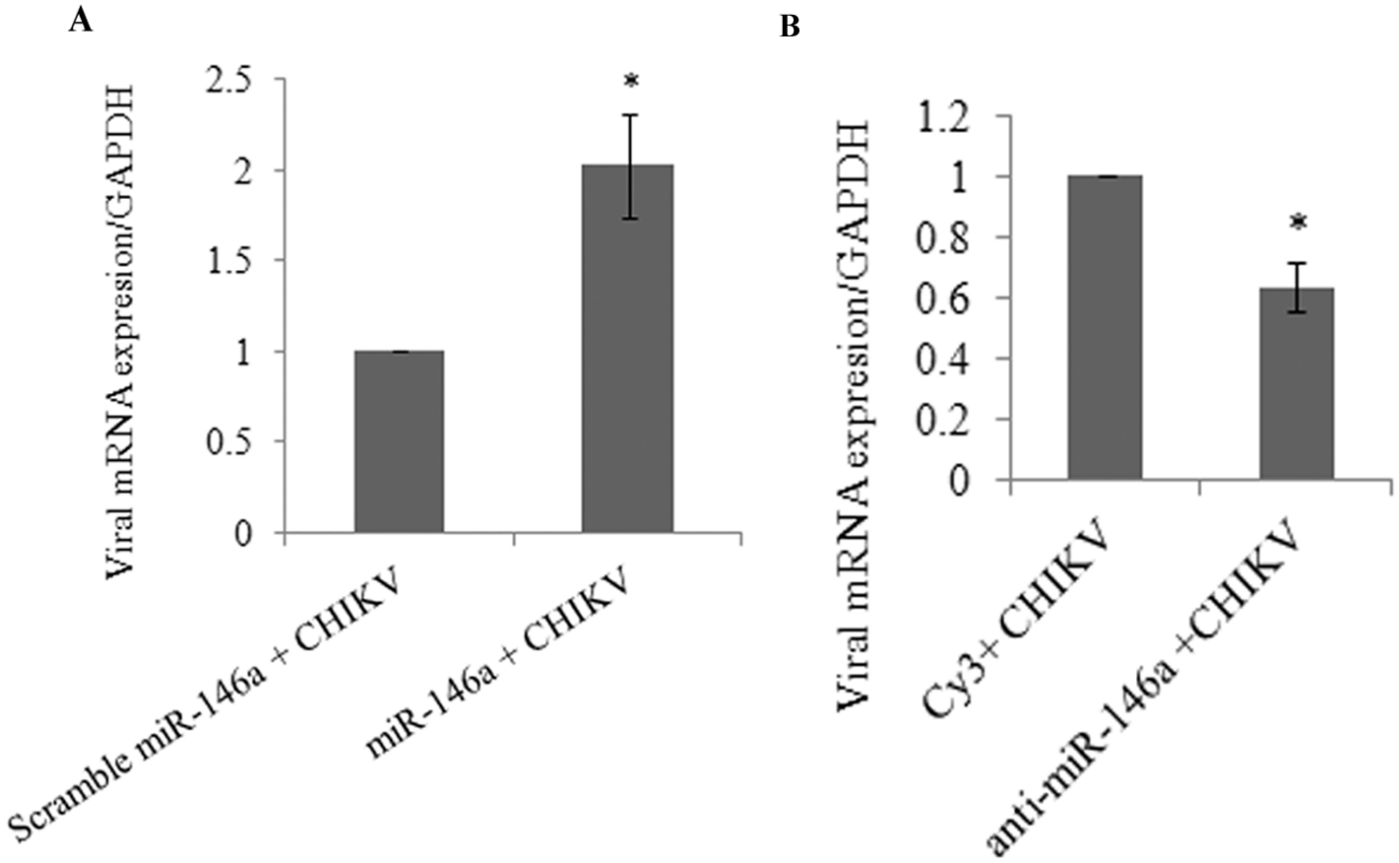
Overexpression and knockdown of miR-146a influences CHIKV replication. miR-146a enhances replication of CHIKV. 24 h post-transfection of scramble miR-146 and miR-146a (Table-1), synovial cells were infected with CHIKV at MOI of 2 and harvested 32 h post infection for RNA isolation. CHIKV transcript levels were quantified via qPCR by using primers against distal 500 bp of 3′UTR region of CHIKV genome, normalized with cellular GAPDH transcript level. (**A**) Graph representing mRNA levels of CHIKV in miR-146a transfected cells as compared to scrambled miR-146a, normalized with GAPDH mRNA levels. (**B**) Graph representing the mRNA levels of CHIKV in cells transfected with anti-miR-146a compared with cy3 labeled controls, normalized with GAPDH transcript level. Three biologically independent experiments were performed and data is presented as mean ± SEM. *p<0.05.

The primary human synovial fibroblasts were transfected with scrambled miR-146a, miR-146a mimics, Cy3 labelled control anti-miR and anti-miR-146a to study the effects of miR-146a on CHIKV replication. Synovial fibroblasts were subjected to same MOI of CHIKV infection (MOI #2) after respective transfections. As compared to scrambled miR-146a, CHIKV replication was found 2 fold higher in miR-146a transfected cells (Figure-7A). However, CHIKV replication was decreased by 40% (Figure-7B) in synovial cells after miR-146a inhibition by anti-miR-146a.

## Discussion

Impaired pro-inflammatory immune responses have been reported in various viral infections [23], [41], [42]. The regulatory role of miRNAs in the regulation of immune responses during viral infections is not well understood [20], [43], [44]. miR-146a is known to be involved in the regulation of immune responses in several viral infections [45], [46], [47]. Elevated levels of miR-146a expression has been reported to impair the expression pattern of IFN-β by targeting TRAF6, in human Dengue virus infected monocytes [23]. Similarly, vesicular stomatitis virus (VSV) has been reported to modulate expression of miR-146a to impair RIG-I signalling and type I interferon production, to inhibit innate antiviral immune response [39]. Interestingly, elevated miR-146a levels have been reported in synovial tissues of rheumatoid arthritis patients [36], [48]. We studied the molecular pathway exploited by CHIKV to modulate the regulation of various adapter molecules TRAF6, IRAK1 and IRAK2 and NF-κB activation in primary human synovial fibroblasts. Upon CHIKV infection in primary human synovial fibroblasts, we observed a significant increase in the levels of expression of miR-146a, which led to the decreased expression levels of TRAF6, IRAK1 and IRAK2; compared to controls (Figure-1A–E). Exogenous over expression of miR-146a in primary human synovial fibroblasts resulted into significant increase in miR-146a (Figure-2A) levels and decreased protein expression levels of TRAF6 (Figure-2B), IRAK1 and IRAK2 (Figure-3). Transfection with scrambled miR-146a did not exhibit any significant change in the levels of expression of miR-146a as well as target genes TRAF6, IRAK1 and IRAK2 (Figure-3). These observations were in accordance with previous reports, on the direct targeting of TRAF6, IRAK1 and IRAK2 by complementary binding between 3′UTR of genes and seed region of miR-146a. Anand Iyer, *et.al* 2012 reported that miR-146a mimics can significantly reduce the mRNA expression levels of TRAF6, IRAK1 and IRAK2 protein levels in IL-1β stimulated human astrocytes and glioblastoma cell line [49]. The induced expression of miR-146a by LMP1 mediated activation of NF-κB during EBV infection in blood cells inhibits NF-κB by down regulation of TRAF6 [25], [40]. Recently, Jin Ho Paik, *et.al* 2011, also reported that miR-146a may function as a tumor-suppressor in NK/T cell lymphoma by down regulating the NF-κB through targeting TRAF6 [50].

To further delineate the specific role of miR-146a in regulation of TRAF6, IRAK1 and IRAK2, inhibitor of miR-146a, referred here as anti-miR-146a was transfected in primary human synovial fibroblasts. Anti-miR-146a has the complementary sequences of miR-146a, which resulted into the sequestration of the mature cellular miR-146a into the synovial fibroblast cells. We observed that the suppression of cellular miR-146a levels (Figure-4A) led to the regain in expression levels of TRAF6, IRAK1 and IRAK2 protein expression levels (Figure-4B, C). The ability of anti-miR-146a in rescuing the expression levels of these proteins were further elucidated by using anti-miR-146a transfected synovial fibroblast cells followed by infection with CHIKV. The expression levels of TRAF6, IRAK1 and IRAK2 were maintained even in presence of CHIKV (Figure-4B, C). These findings clearly demonstrated that the CHIKV mediated suppression of TRAF6, IRAK1 and IRAK2 occurs through upregulation of miR-146a in primary human synovial fibroblasts.

Cytokine signalling through TRAF6, IRAK1 and IRAK2 leads to activation of NF-κB pathway. This triggers the translocation of NF-κB into nucleus and transcriptional activation of set of genes critical for immune response and inflammatory events [45]. To emphasize the specificity and exploitation of miR-146a as negative feedback tool for suppression of NF-κB activity by CHIKV, we studied the activation of NF-κB in CHIKV infected primary human synovial fibroblasts. Reduced phosphorylation levels of NF-κB (p65) and decreased NF-κB activity were observed in CHIKV infected synovial fibroblasts respectively (Figure-5A, Figure-6A).

Similarly, miR-146a over expression led to the decreased phosphorylated form of NF-κB (p65); compared to scramble miR-146a transfected cells (Figure-5C, D). Decreased luciferase activity was observed in miR-146a overexpressed cells in NF-κB promoter assay (Figure-6B). These findings confirmed that the regulation of NF-κB activation is mediated via miR-146a in CHIKV infected human synovial fibroblasts. NF-κB promoter assay showed restored levels of NF-κB activity in the synovial fibroblasts transfected with anti-miR-146a (Figure-6C). Significantly an enhanced level of activation of NF-κB was observed even in CHIKV infected cells at same MOI (Figure-6C). These results were compared with down regulation of NF-κB promoter activity in CHIKV infection to highlight the role of cellular miR-146a in maintaining the NF-κB activity. We demonstrated that CHIKV exploits the negative regulatory loop, in which early NF-κB activation induces miR-146a expression; which results into down-regulation of TRAF6, IRAK1 and IRAK2 to further restrain the activity of NF-κB [45]. Negative regulatory function of miR-146a has been reported in estrogen treated splenic lymphocytes; where decrease in miR-146a led to increase in LPS induced IFN-γ and iNOS production through TLR signalling [51]. VSV infections have also been reported to utilize negative feedback pathway of miR-146a to regulate type I interferon production [39]. A general induction of the nuclear transcription factor κB (NF-κB) plays an essential role in stimulating the expression of inflammatory genes; which are particularly involved in the progression of inflammatory diseases like arthritis [52]. The general repression of NF-κB activity as well as defective type I interferon response through the targeting of common adapter molecules TRAF6, IRAK1, IRAK2 by CHIKV explains the susceptibility of neonates and elderly (low immune strength) for CHIKV infection [4], [10]. Our results showed an enhanced rate of CHIKV replication in presence of higher level of miR-146a (miR-146a overexpression). In contrast, when cellular miR-146a levels were inhibited by application of anti-miR-146a, CHIKV replication decreased (Figure-7), which explained the exploitation of miR146a mediated repression of NF-κB activity in favour of increasing viral replication and aggravating pathological manifestations during CHIKV infections. The expression of inflammatory cytokines (TNF-α, IL6 and IL-1β) (Table-2) decreased in miR-146a overexpressing cells. In contrast the expression of inflammatory cytokines was induced in anti-miR-146a transfected cells (Figure S1). This indicated a general suppression of NF-κB mediated cytokine generation in CHIKV infected synovial fibroblasts, which might be a strategy utilized by CHIKV to facilitate their replication.

**Table 2:** List of Primers.

Name	Forward (5′-3′)	Reverse (5′-3′)
GAPDH	ATGGGGAAGGTGAAGGTCG	GGGGTCATTGATGGCAACAATA
CHIKV	ATGCCCATCTCCATCGACATAC	ATATACCTTCATACTTAATTGTC
TNF-α	CCTCTCTCTAATCAGCCCTCTG	GAGGACCTGGGAGTAGATGAG
IL-6	ACTCACCTCTTCAGAACGAATTG	CCATCTTTGGAAGGTTCAGGTTG
IL-1β	AGCTACGAATCTCCGACCAC	CGTTATCCCATGTGTCGAAGAA

**Figure 8:**
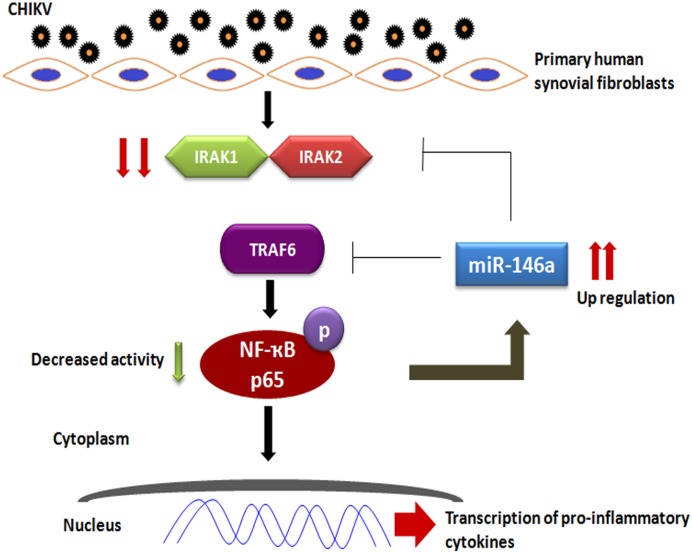
CHIKV mediated regulation of NF-κB by miR-146a modulation in primary human synovial fibroblasts. CHIKV infection induces the expression of cellular miR-146a in primary human synovial fibroblasts, which in turn downregulates the expression of TRAF6, IRAK1 and IRAK2. Decreased expression of these immune modulators results into reduced NF-κB phosphorylation and activation in primary human synovial fibroblasts.

In summary, our study confirms that, CHIKV induces the expression levels of miR-146a in primary human synovial fibroblasts; which in turn suppresses TRAF6, IRAK1, IRAK2 expression levels and downstream NF-κB activity through negative feedback loop (Figure-8). CHIKV utilizes the miR-146a, as a negative regulator of general antiviral response. This study provides an understanding about the immune response of human synovial fibroblasts in CHIKV infection and insights into immune evasion strategies adapted by CHIKV.

## Supporting Information

Figure S1 Overexpression and knockdown of miR-146a affects TNF-α, IL-6 and IL-1β transcript level. miR-146a suppress the transcript levels of TNF-α, IL-6 and IL-1β upon CHIKV infection (Table-2). **(A)** Graph bar representing the mRNA levels of TNF-α in synovial fibroblast cells upon overexpression and knockdown of miR-146a. **(B)** Graph bar showing changes in transcript level of IL-6 in synovial fibroblast cells upon overexpression and knockdown of miR-146a. **(C)** Bar diagram indicating the changes in transcript level of IL-1β upon overexpression and knockdown of miR-146a in primary synovial fibroblast cells. Cytokine transcript level detection was done by qPCR normalized with GAPDH transcript level (Table-2). All the experiments were repeated three times and results shown as mean ± SEM.
(TIF)
