# Supplementary figures and images for: Chikungunya Virus Exploits miR-146a to Regulate NF-κB Pathway in Human Synovial Fibroblasts
Source: PLoS One. 2014 Aug 1;9(8):e103624. doi: 10.1371/journal.pone.0103624 (PMC4118904; doi:10.1371/journal.pone.0103624)

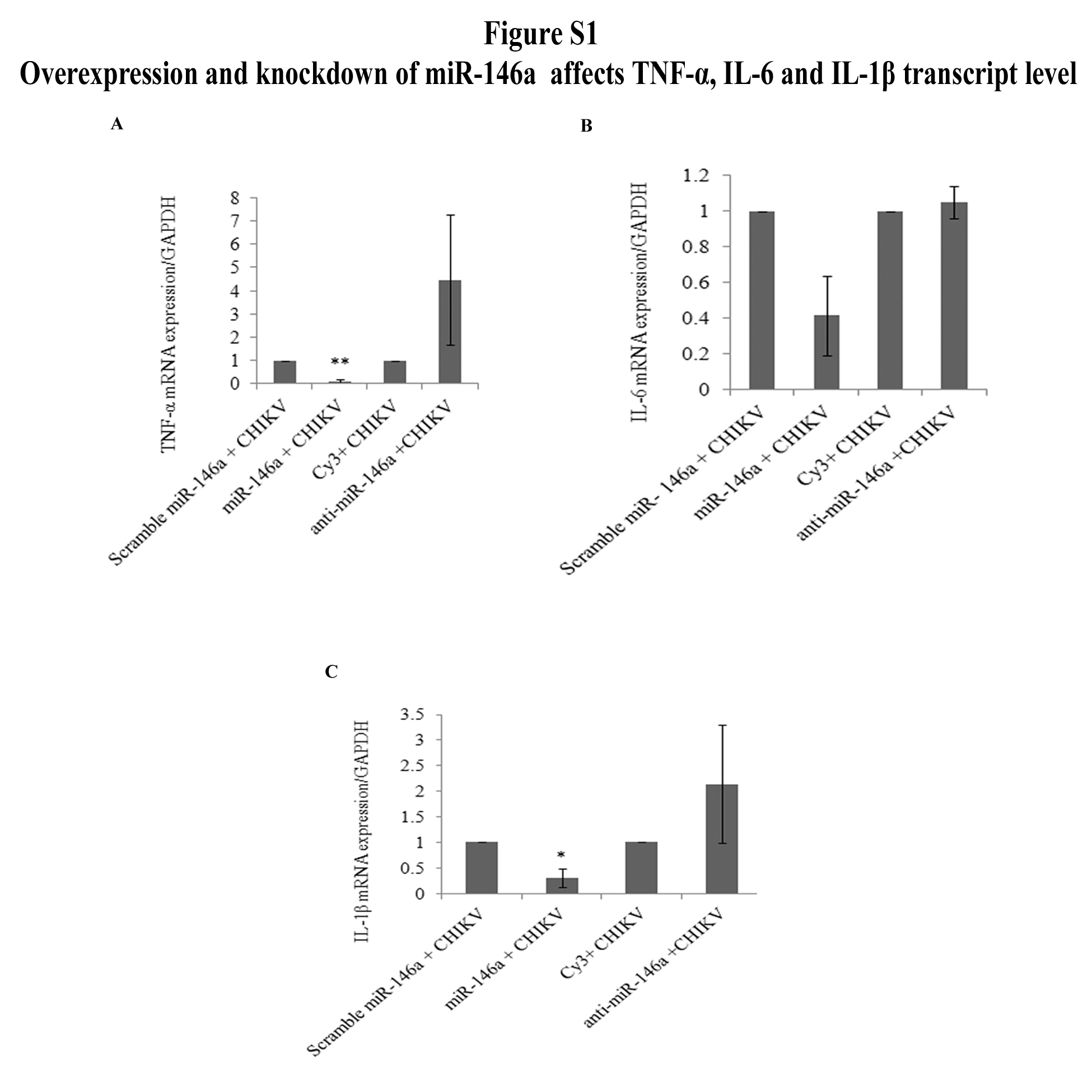

Supplement: Figure S1 — Overexpression and knockdown of miR-146a affects TNF-α, IL-6 and IL-1β transcript level. miR-146a suppress the transcript levels of TNF-α, IL-6 and IL-1β upon CHIKV infection (Table-2). (A) Graph bar representing the mRNA levels of TNF-α in synovial fibroblast cells upon overexpression and knockdown of miR-146a. (B) Graph bar showing changes in transcript level of IL-6 in synovial fibroblast cells upon overexpression and knockdown of miR-146a. (C) Bar diagram indicating the changes in transcript level of IL-1β upon overexpression and knockdown of miR-146a in primary synovial fibroblast cells. Cytokine transcript level detection was done by qPCR normalized with GAPDH transcript level (Table-2). All the experiments were repeated three times and results shown as mean ± SEM. (TIF) [file pone.0103624.s001.tif]
